# PhcrTx2, a New Crab-Paralyzing Peptide Toxin from the Sea Anemone *Phymanthus crucifer*

**DOI:** 10.3390/toxins10020072

**Published:** 2018-02-07

**Authors:** Armando Alexei Rodríguez, Anoland Garateix, Emilio Salceda, Steve Peigneur, André Junqueira Zaharenko, Tirso Pons, Yúlica Santos, Roberto Arreguín, Ludger Ständker, Wolf-Georg Forssmann, Jan Tytgat, Rosario Vega, Enrique Soto

**Affiliations:** 1 Core Facility Functional Peptidomics, Ulm University Medical Center, Albert-Einstein-Allee 47, 89081 Ulm, Germany; ludger.staendker@uni-ulm.de; 2 Department of Experimental and Clinical Peptide Chemistry, Hannover Medical School (MHH), Feodor-Lynen-Straße 31, D-30625 Hannover, Germany; wg.forssmann@pharis.de; 3 Centro de Bioproductos Marinos (CEBIMAR), Loma y 37, Alturas del Vedado, Habana CP 10600, Cuba; agarateix@gmail.com; 4 Instituto de Fisiología, Benemérita Universidad Autónoma de Puebla, 14 sur 6301, CU, San Manuel, Puebla CP 72750, Mexico; emilio.salceda@gmail.com (E.Sa.); axolotl_56@yahoo.com.mx (R.V.); esoto24@gmail.com (E.So.); 5 Toxicology & Pharmacology, University of Leuven (KU Leuven), Campus Gasthuisberg O&N2, Herestraat 49, P.O. Box 922, 3000 Leuven, Belgium; Steve.Peigneur@pharm.kuleuven.be (S.P.); Jan.Tytgat@pharm.kuleuven.be (J.T.); 6 Laboratory of Genetics, Butantan Institute, São Paulo 05503-900, Brazil; a.j.zaharenko@gmail.com; 7 Centro Nacional de Biotecnología (CNB-CSIC), Departamento de Inmunología y Oncología, C/Darwin 3, Campus de Cantoblanco, 28049 Madrid, Spain; tpons@cnb.csic.es; 8 Department of Plant Pathology, Citrus Research and Education Center, University of Florida, Lake Alfred, FL 33850, USA; yulica_santos@yahoo.com; 9 Instituto de Química, Universidad Nacional Autónoma de México, Delegación Coyoacán, Ciudad de México 04510, Mexico; arrespin@gmail.com

**Keywords:** sea anemone, neutoxin, glutamate receptor, defensin-like fold, ion channels, *Phymanthus crucifer*

## Abstract

Sea anemones produce proteinaceous toxins for predation and defense, including peptide toxins that act on a large variety of ion channels of pharmacological and biomedical interest. *Phymanthus crucifer* is commonly found in the Caribbean Sea; however, the chemical structure and biological activity of its toxins remain unknown, with the exception of PhcrTx1, an acid-sensing ion channel (ASIC) inhibitor. Therefore, in the present work, we focused on the isolation and characterization of new *P. crucifer* toxins by chromatographic fractionation, followed by a toxicity screening on crabs, an evaluation of ion channels, and sequence analysis. Five groups of toxic chromatographic fractions were found, and a new paralyzing toxin was purified and named PhcrTx2. The toxin inhibited glutamate-gated currents in snail neurons (maximum inhibition of 35%, IC_50_ 4.7 µM), and displayed little or no influence on voltage-sensitive sodium/potassium channels in snail and rat dorsal root ganglion (DRG) neurons, nor on a variety of cloned voltage-gated ion channels. The toxin sequence was fully elucidated by Edman degradation. PhcrTx2 is a new β-defensin-fold peptide that shares a sequence similarity to type 3 potassium channels toxins. However, its low activity on the evaluated ion channels suggests that its molecular target remains unknown. PhcrTx2 is the first known paralyzing toxin in the family Phymanthidae.

## 1. Introduction

Sea anemones produce a large number of proteinaceous toxins for preying on small crustaceans and fishes, and for defense against predators [1,2]. These toxins comprise mainly cytolysins, protease inhibitors, and a large variety of peptides acting on sodium or potassium channels; more recently, acid-sensing ion channel (ASIC) and TRPV1 channels toxins have also been discovered [1]. These peptide toxins are valuable pharmacological tools for studying the structure and function of ion channels [2], which are involved in many physiological and pathological processes. Ion channels constitute a primary site of action for many antiepileptic drugs, local anesthetics, migraine treatments, antipsychotics and mood stabilizers, antiarrhythmics, antihypertensives, and oral hypoglycemic agents [3].

The discovery of novel families of sea anemone peptide toxins [4,5,6] and the development of “omics” studies [7,8,9,10,11,12,13,14] have revealed a large diversity of peptides in several species. In addition, novel molecular scaffolds and post-translational modifications in sea anemone peptides have been described [4,6,9,15]. However, the number of known sea anemone peptide toxins is still small, considering the large variety of peptides expected from their whole peptidomes, and taking into account that only a minor fraction of the total number of known species has been analyzed. Therefore, many new peptide toxins or even new families of peptide toxins are expected to be discovered from these organisms.

An approach often used for the discovery of sea anemone toxins is based on a well-known crab bioassay [16,17,18], due to the sensitivity of crustaceans to sea anemone toxins. Crab bioassay-guided purifications have allowed the discovery of more than 30 sea anemone toxins, most of them belonging to different groups of sodium channel toxins [16,19,20,21,22,23,24,25,26,27]. A fewer number of peptides acting on potassium channels, and also on targets to be elucidated, have been found by observations of toxicity signs induced by injection to crabs. Such peptides remain to be functionally characterized on a variety of tentative targets, not only voltage-gated ion channels, but also ligand-gated ion channels [2,21,28], such as glutamate-gated ion channels. Glutamate is the excitatory neurotransmitter in the crustacean neuromuscular junction [29]; therefore, it has been suggested that glutamate receptor antagonists could also be found among sea anemone toxins [30]. However, to date, no peptide acting on glutamate-gated ion channels has been characterized from sea anemones, in contrast with other venomous animals such as cone snails [31] and spiders [32]. *Phymanthus crucifer* (Le Sueur, 1817) is a species of sea anemone that commonly inhabits the Caribbean Sea. This species is known to produce a large diversity of peptides [33]; however, only one peptide toxin has been characterized, PhcrTx1, which is an acid-sensing ion channel inhibitor presenting an inhibitor cystine knot (ICK) motif [6]. In this work, we performed a crab bioassay-guided chromatographic fractionation of the aqueous extract obtained from the sea anemone *P. crucifer*. Several chromatographic fractions showing crab-paralyzing activity were isolated, and a major paralyzing peptide (PhcrTx2) was purified and sequenced. This toxin showed sequence similarity to defensin-like peptides that exhibit a variety of biological activities, including crab toxicity and voltage-gated (sodium or potassium) channel inhibition. PhcrTx2 represents the first known member of the defensin-fold family of polypeptides isolated from the family Phymanthidae. This is the first study describing paralyzing toxins in this sea anemone family.

## 2. Results

### 2.1. Bioassay-Guided Purification of P. crucifer Toxins

**Figure 1:**
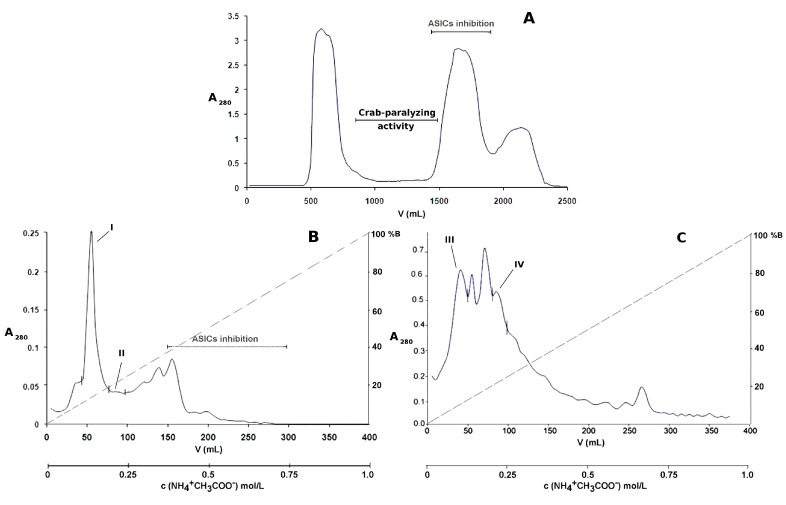
(**A**) Gel filtration profile of *P. crucifer* aqueous extract. The soluble material contained in 5 grams of whole-body homogenate (350 mg/90 mL) was fractionated on Sephadex G-50 (5 × 93 cm) at 2 mL/min using 0.1 mol/L ammonium acetate. Fractions of 20 mL each were collected; those within the elution volumes of 820 mL to 1460 mL were paralyzing to all of the crabs, and were pooled; (**B**) Cation-exchange chromatographic profile of the crab-paralyzing pool of chromatographic fractions from Sephadex G-50, in Fractogel EMD SO_3_^−^ 650 M (1.8 × 5 cm); (**C**) Anion-exchange chromatographic profile of the non-retained fraction from the cation exchanger, in Fractogel EMD DEAE 650 M (1.8 × 5 cm). Both separations (**B**,**C**) were done at a flow rate of 1 mL/min using a 400-mL gradient, from 0.01 mol/L to 1 mol/L ammonium acetate. Eighty fractions of 5 mL each were collected in every chromatographic separation. Dashed lines in the ion-exchange chromatographic profiles represent the gradient of ammonium acetate. Fractions exhibiting toxicity to crabs were named I, II, III, and IV. The pools of fractions that inhibited acid-sensing ion channels are shown in both gel-filtration and cation-exchange chromatographic profiles, according to previous results with the same *P. crucifer* homogenate, using identical conditions [6]. PhcrTx1, an acid-sensing ion channel toxin from *P. crucifer* [6], eluted inhibiting pools of chromatographic fractions in the ASICs, as shown in (**A**,**B)**. As shown, the crab-paralyzing zone and the ASICs inhibition zone barely overlapped in the gel filtration profile (**A**); and completely separated from each other in the cation-exchange profile (**B**). PhcrTx1 is not present among the crab-paralyzing chromatographic fractions isolated from the ion-exchange chromatographic separations.

The aqueous extract (350 mg/90 mL 0.1 mol/L ammonium acetate) obtained from 5 g of the sea anemone homogenate showed crab-paralyzing activity, and was subjected to chromatographic fractionation. Sephadex G-50 is a low-pressure medium that has been commonly used for the group fractionation of complex samples such as sea anemone extracts, due to its suitable fractionation range (1.5 kDa to 30 kDa) for the separation of the peptide fraction from proteins, and low molecular weight compounds. The sample was fractionated by gel filtration on Sephadex G-50 M, and aliquots were taken from every other fraction (every 20 mL) for toxicity screening purposes. Due to the large number of crabs needed for determining the ED_50_ or LD_50_ of every toxic chromatographic fraction, and the unavailability of such a number, we focused our efforts on characterizing the toxicity of only pure compounds submitted to both chemical and pharmacological characterization. Unless stated otherwise, three crabs were used per sample at the dose of 2000 μg/kg for biological activity screening after every chromatographic step, and only those fractions that paralyzed all of the crabs were selected for further purification/analysis. The crab-paralyzing activity was detected in the broad zone (elution volume 820–1460 mL) indicated in the chromatographic profile (Figure 1A). The low UV absorption of the crab-paralyzing fractions from Sephadex G-50 at 280 nm (Figure 1A) indicated that these are composed of low abundance peptides and proteins distributed within the fractionation range of Sephadex G-50. In addition, the low efficiency and resolution of a low-pressure medium such as Sephadex G-50 produces broad overlapping peaks, which disfavors the appearance of any notable signals coming from these low abundance molecules. As a result, these crab-paralyzing molecules, distributed within a large elution volume range, yield a flat low UV absorption zone in the chromatographic profile. On the other hand, the crab-paralyzing zone is located between two high intensity signals whose components are mostly located out of the fractionation range of the column. Molecules eluting out of the fractionation range (>30 kDa or <1.5 kDa), specifically near the void volume (550 mL) or the total volume of the column (1825 mL), tend to be poorly resolved or not separated at all. Therefore, their contributions to the UV absorption add up to yield a significant increase of signal intensity at both sides of the crab-paralyzing zone in the Sephadex G-50 chromatographic profile.

The toxic fractions were pooled and applied to a Fractogel EMD SO_3_^−^ 650 M cation-exchange column (Figure 1B), and the non-retained fraction was subsequently applied to a Fractogel EMD DEAE 650 M anion-exchange column (Figure 1C). For screening purposes, small aliquots were pooled according to the following groups: fractions from 0–50 mL, 50–100 mL, 100–150 mL, 150–200 mL, 200–250 mL, 250–300 mL, 300–350 mL, and 350–400 mL elution volume. Only the pools 0–50 mL and 50–100 mL, which were from cation-exchange chromatography and anion-exchange chromatography, respectively, paralyzed all of the crabs. Then, every single fraction (1 to 20) from these pools was assayed, and those paralyzing all of the crabs were pooled as I, II, III, and IV (Figure 1B,C).

**Figure 2:**
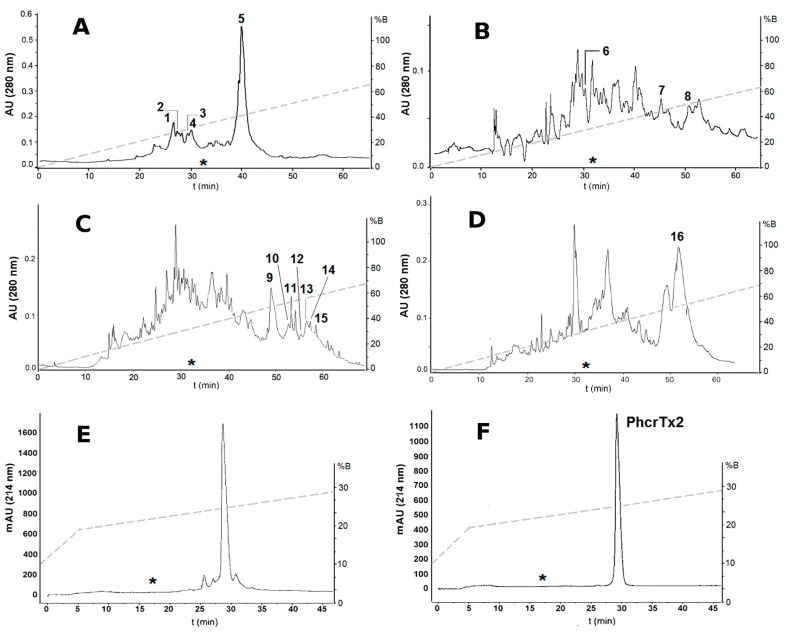
Reversed-phase chromatographic profiles of crab-paralyzing fractions from ion-exchange chromatography. (**A**,**B**) Reversed-phase chromatographic profiles of fractions I and II previously separated from cation-exchange chromatography, respectively; (**C**,**D**) Reversed-phase chromatographic profiles of fractions III and IV previously separated from anion-exchange chromatography, respectively. Conditions: Hypersil H5 ODS column (4.6 × 250 mm), flow rate 0.8 mL/min, linear gradient from 0 to 80% B in 80 min. Chromatographic fractions showing toxicity to crabs are indicated in the figure (1 to 16); (**E**,**F**) Reversed-phase chromatographic purification of fraction number 5. Conditions: Discovery RPC18 HPLC column (4.6 × 250 mm), flow rate of 1 mL/min, gradient from 10 to 20% B in 5 min, followed by 20 to 30% in 50 min. The pure toxin was named PhcrTx2. The dashed line in every chromatographic profile represents the gradient of acetonitrile. The asterisk (*) in (**A**–**F**) represent the point where PhcrTx1 elutes in the same chromatographic conditions, according to previous results [6].

The crab-paralyzing fractions (I, II, III, and IV) from ion-exchange chromatography were subsequently subjected to reversed-phase C18 HPLC (Figure 2A–D). The chromatographic fractions from RPC18-HPLC were pooled according to peak shape, and assayed on crabs. A total of 16 toxic reversed-phase chromatographic fractions were separated and analyzed by MALDI-TOF-MS. These toxic fractions were classified into five groups according to their chromatographic behavior, molecular masses, and paralyzing effects on crabs (Table 1, Supplementary Table S1). A major reversed-phase fraction (number 5 in Figure 2A) was subjected to repurification on an analytical reversed-phase C18 column (Figure 2E,F), and the pure toxin was named PhcrTx2. The amount of pure peptide was 420 μg, which represents the 0.0084% of 5 g *P. crucifer* freeze-dried whole homogenate.

In a previous work [6], PhcrTx1, an acid-sensing ion channel toxin, was isolated using the same conditions of gel filtration chromatography, cation-exchange chromatography, and reversed-phase chromatography. Figure 1A shows the crab-paralyzing zone of the gel filtration chromatographic profile, which almost completely excludes the ASICs inhibition zone from which PhcrTx1 was previously isolated [6]. Also, Figure 1B shows that the crab-paralyzing zone (pools I and II) is totally separated from the ASICs inhibition zone where PhcrTx1 was eluted [6]. Therefore, at this point after the cation-exchange chromatography, PhcrTx1 and PhcrTx2 are completely separated. Figure 2A–F show the retention time (*) of PhcrTx1 in similar conditions of reversed-phase chromatography, indicating that PhcrTx2 is more strongly retained than PhcrTx1, in contrast with its behavior in cation-exchange chromatography (pool I, Figure 1B), where it elutes much earlier than PhcrTx1 (ASICs inhibition, Figure 1B).

### 2.2. Biological Evaluation of PhcrTx2

#### 2.2.1. Crab Toxicity Assay

PhcrTx2 was evaluated by injecting several doses (62.5 µg/kg, 125 µg/kg, 250 µg/kg, 500 µg/kg, 1000 µg/kg, 2000 µg/kg, and 4000 µg/kg) to groups of six crabs. The number (and percentage) of paralyzed crabs was 0 (0%), 0 (0%), 1 (16.7%), 2 (33.3%), 4 (66.7%), 5 (83.3%), and 6 (100%) out of 6, for every mentioned dose, respectively. The toxin was not lethal, but only paralyzing, with an ED_50_ = 707 µg/kg (see Supplementary Figure S1). The onset of the tetanic paralysis was observed after 30 min of toxin administration. After two hours, the crabs remained upward facing and showing uncoordinated movements of legs.

#### 2.2.2. Effects of PhcrTx2 on Native Na_v_, K_v_ and Glutamate-Gated Currents

In the toxicity assays performed on crabs *Uca thayeri*, PhcrTx2 was not lethal but only paralyzing, indicating that it is most probably not acting on Na_v_, taking into account that Na_v_ sea anemone toxins are lethal to crabs. For example, toxins RpI, II, III, and IV (from *Radianthus* *paumotensis*), are lethal to crabs, with an LD_50_ in the range between 10 μg/kg and 90 μg/kg, [26]; AETX II and III (from *Anemonia erythraea*) have LD_50_ values of 0.53 μg/kg and 0.28 μg/kg, respectively [16]; Am I (from *Antheopsis maculata*) has an LD_50_ of 830 mg/kg [21]. Also, the toxicity signs induced by PhcrTx2 differ from those caused by Na_v_ toxins, by its late appearance and lower intensity. Hence, considering that crustaceans have a glutamatergic neuromuscular junction, and they are habitual preys of sea anemones, we evaluated the glutamate-induced responses in snail neurons.

Additionally, given that many sea anemone (including crab-paralyzing) toxins show activity on mammalian ion channels, mainly by inhibiting voltage-gated potassium channels [34,35] or by delaying the inactivation of voltage-gated sodium channels [36,37], we carried out an additional set of experiments to evaluate the PhcrTx2 action on voltage-activated Na^+^ and K^+^ currents in rat dorsal root ganglion (DRG) neurons. Although some sea anemone peptides have been found to be ASIC toxins [4,6,38], PhcrTx2 is unlikely to have activity on acid-sensing ion channels from rat dorsal root ganglion neurons. The updated Figure 1B shows that the crab-paralyzing zone (where PhcrTx2 elutes) and the ASIC inhibition zone (where PhcrTx1 eluted in identical conditions [6]) in the cation-exchange chromatography are completely separated. Therefore, PhcrTx2 and the other crab-paralyzing basic peptides are not likely to exhibit ASIC inhibitory activity. Therefore, PhcrTx2 was not evaluated on these ion channels.

##### PhcrTx2 Evaluation on Snail Neurons 

**Figure 3:**
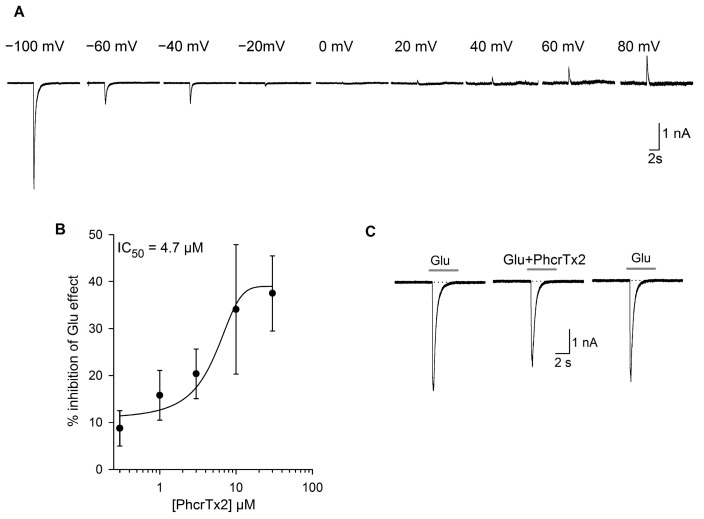
Effect of PhcrTx2 on a glutamate-evoked current in isolated snail neurons. (**A**) Glutamate-evoked (at 1 mM of Glu) currents registered at different holding potentials (from −100 mV to +80 mV) show a current reversal at about 0 mV, indicating that it most probably is a non-selective cation permeant channel; (**B**) Concentration-response relationship of the inhibitory effect of PhcrTx2 on glutamate-gated currents. Data were fitted by a dose-response function with an IC_50_ of 4.7 µM. Each point represents the mean ± SE from four to seven neurons; (**C**) Representative current traces elicited by glutamate (1 mM) under control condition, in the presence of 30 µM PhcrTx2, and after washout of the toxin.

Recordings of the glutamate-evoked currents were performed on cultured isolated neurons from the land snail *Helix aspersa*. The neurons (*n* = 27) had a capacitance of 55 ± 14.6 pF, corresponding to an approximate cell diameter of 24–53 μm (mean = 42 ± 6 μm). In snail neurons voltage-clamped at negative membrane potentials (−100 mV), glutamate 1 mM (5 s) produced an inward current that shows a fast activation, followed by a desensitization phase that could have a variable steady-state component. We carried out a group of experiments to study glutamate-evoked currents in snail neurons (*n* = 5). For this purpose, 1-mM glutamate currents were elicited at different membrane potentials from −100 mV to +80 mV (Figure 3A); the largest current was obtained at a holding potential of −100 mV (average values about 2.6 ± 1.4 nA). The current had an average reversal potential close to 0 mV, from which the current increased until reaching a value of 0.3 ± 0.1 nA at 80 mV. The dose responses curves and all of the results that are shown and discussed in this article were consequently done at a holding potential of −100 mV.

The reversal potential of the glutamate-evoked currents was around zero (Figure 3A). The sustained application of PhcrTx2 produced a significant decrease of the peak current (*p* ≤ 0.05, Student’s *t*-test) in the concentration range between 3 μM and 30 μM (*n* = 23). The inhibitory effect was concentration-dependent (Figure 3B). The concentration-response relationship had an IC_50_ of 4.7 µM. At 3 µM, the closest value to the IC_50_ tested, the inhibition was statistically significant (*p* ≤ 0.05, Student *t*-test), and it was about 20.4 ± 5.3% (*n* = 8, *p* = 0.03), while the maximum inhibition observed at the highest concentration (30 µM) was about 37.5 ± 8.1% (*n* = 7, *p* = 0.01). The inhibitory effect on the peak current was very fast, reaching stability within about 2 min, and was fully reversible after washing the preparation (between the first and the second minute, Figure 3C). The sustained component and the current desensitization rate were not significantly affected at any toxin concentration tested; for example, at 3 µM, the τ_des_ in control was 174.7 ± 50.6 ms, whereas τ_des_ in the presence of a toxin was 249.2 ± 60.5 ms, *p* > 0.05, Student *t*-test, *n* = 9. 

An evaluation of the effect of PhcrTx2 on voltage-gated K^+^ currents in snail isolated cells (*n* = 29) in the concentration range from 3 µM to 30 µM showed the effects to be highly variable, and did not reach statistical significance. For example, 10 µM of toxin produced a significant decrease in the K^+^ current amplitude at the peak and at the steady state of 13.9 ± 2.1% (*p* = 0.001) and 16.0 ± 4.8% (*p* = 0.002), respectively. However, the effect at 30 µM was significant neither on the peak current nor on the steady state current.

##### PhcrTx2 Evaluation on Rat DRG Neurons

The action of PhcrTx2 was also evaluated on the voltage-dependent Na^+^ current in DRG neurons (Supplementary Figure S2A). The toxin caused a decrease in the amplitude of the peak current at concentration values between 1–10 µM, with an IC_50_ of 0.9 ± 0.2 µM (Supplementary Figure S2B). The maximal effect of the toxin on the Na^+^ current was 16%; this action was partially reversible after washout (≈90%). No significant effect was observed on the time course of inactivation for any of the concentrations studied. 

The perfusion of cells with 30 µM of PhcrTx2 while recording K^+^ currents produced an inhibition of the K^+^ currents that was concentration-dependent, with an IC_50_ = 6.4 ± 0.2 µM and 8.2 ± 0.7 µM for the peak current and steady-state current, respectively (*n* = 31; Supplementary Figure S2C–F). The maximum inhibitory effect was of 26.9 ± 4.1% in the peak current and 41.4 ± 4.8% in the steady-state current when PhcrTx2 30 μM was perfused. This effect was partially reversible (82%) after repeated washing of the preparation. 

#### 2.2.3. Effects of PhcrTx2 on Cloned Voltage-Gated Ion Channels 

At a concentration of 5 µM, PhcrTx2 was investigated for its activity on 10 different K_v_ channel isoforms and five different Na_v_ channel isoforms expressed in *X. laevis* oocytes. PhcrTx2 was tested against members of the Shaker (K_v_1.1, K_v_1.2, K_v_1.3, K_v_1.4, K_v_1.6, and Shaker IR), Shab (K_v_2.1), Shaw (K_v_3.1), Shal (K_v_4.2), and Eag (K_v_10.1) K_v_ subfamilies. Furthermore, the toxin was also tested on Na_v_ channel isoforms Na_v_1.4, Na_v_1.5, Na_v_1.6, Na_v_1.8, and DmNa_v_1. No effect was observed for any of the investigated channels (Supplementary Figure S3 and Supplementary Table S2).

### 2.3. PhcrTx2 Sequence and Computational Analysis

**Figure 4:**
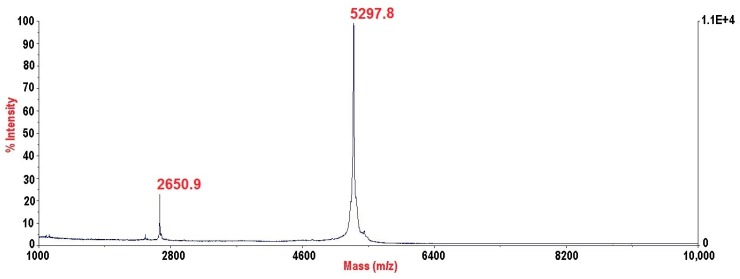
MALDI-TOF mass spectrum of PhcrTx2. An intense signal of *m*/*z* 5297.8 from the monoprotonated peptide ion [M + H]^+^ was detected, corresponding to a molecular mass of 5296.8 Da.

PhcrTx2 was subjected to automated N-terminal degradation, yielding a full sequence of 46 amino acid residues, ^1^ALPCRCEGKTEYGDKWIFHGGCPNDYGYNDRCFMKPGSVCCYPKYE^46^. The protein sequence data reported in this paper appears in the UniProt Knowledgebase under the accession number C0HK75. Its theoretical average molecular mass of 5296.9 Da (assuming the formation of three disulfide bridges) is very close to the experimental value of 5296.8 Da (Figure 4), as determined by MALDI-TOF-MS.

**Figure 5:**
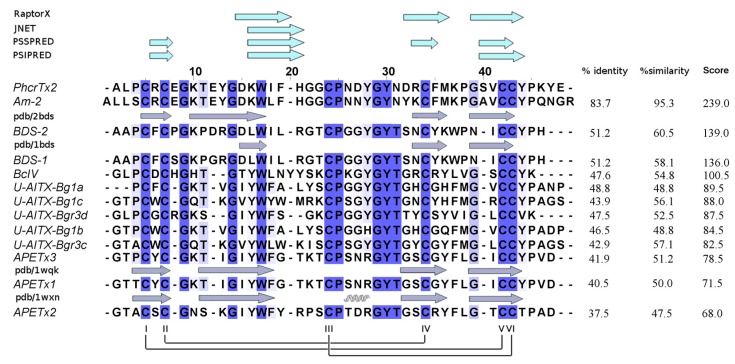
Multiple sequence alignment (MAFF-LINSi) of PhcrTx2 and related sequences listed in order of descending score, according to pairwise sequence alignments using the Smith–Waterman algorithm (EMBOSS water tool). Dark blue columns represent the alignment of identical amino acid residues, whereas the lighter colors represent the alignment of less conserved positions. The secondary structure features from experimental 3D structures are represented above their corresponding sequences. The PhcrTx2-related sequences correspond to the sea anemone toxins Am-2 (UniProtKB P69930), BDS-2 (UniProtKB P59084), BDS-1(UniProtKB P11494), BcIV (UniProtKB P84919), U-AITX-Bg1a (UniProtKB G0W2H7), U-AITX-Bg1c (UniProtKB G0W2H9), U-AITX-Bg3d (UniProtKB G0W2I1), U-AITX-Bg1b (UniProtKB G0W2H8), U-AITX-Bg3c (UniProtKB G0W2I0), APETx3 (UniProtKB B3EWF9), APETx1 (UniProtKB P61541), and APETx2 (UniProtKB P61542). The upper side of the figure shows the secondary structure elements predicted for PhcrTx2 using RaptorX, JNET, PSSPRED, and PSIPRED. The lower side of the figure shows the representative disulfide bridge pattern of the family Defensin 4, which is Cys5-Cys42, Cys7-Cys34, and Cys24-Cys43 in the multiple sequence alignment. The same S-S arrangement is expected for PhcrTx2, corresponding to Cys4-Cys40, Cys6-Cys32, and Cys22-Cys41 in its sequence, which fits the pattern I-V, II-IV, III-VI found in β-defensins.

The theoretical isoelectric point of PhcrTx2 is 7.61, indicating that it is a slightly basic peptide. PSI-BLAST results showed significant sequence similarity to several sea anemone toxins already annotated in non-redundant NCBI (nrNCBI) and UniProt/Swiss-Prot databases: (a) Am II (83.7% identity), a crab-paralyzing toxin from *Antheopsis maculata* [21]; and (b) BDS-I and BDS-II (both, 51.2% identity), which are antiviral and antihypertensive toxins that inhibit K_v_3 channels, and delay the inactivation of Na_v_ channels [34,35,39], which were isolated from *Anemonia viridis*. These toxins are classified within the “Defensin 4” protein family (Pfam accession: PF07936), to which PhcrTx2 is closely related, according to the search against the Pfam database [40]. PF07936 is composed of sea anemone neurotoxins BDS-I, BDS-II, APETx1 [41,42], and APETx2 [38,43], which are defensin-like folded peptides, and representative members with three-dimensional (3D) structures determined by NMR. Figure 5 shows a multiple sequence alignment of PhcrTx2 and the PF07936 members of highest similarity, according to the EMBOSS Water tool. The multiple sequence alignment indicated that PhcrTx2 has the disulfide bonding pattern Cys4-Cys40, Cys6-Cys32, and Cys22-Cys41. The toxin fits very well into the features described for β-defensins [44] such as (1) composed of 35–50 amino acid residues; (2) contain six cysteine residues linked according to the connectivity I-V, II-IV, III-VI; and (3) the last two cysteines are consecutively situated (in a CCXn pattern where *n* >1) near the C-terminus.

**Figure 6:**
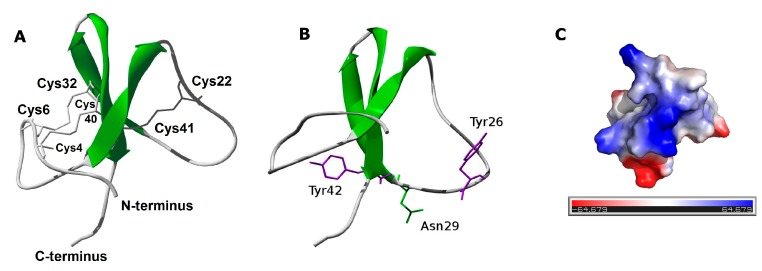
(**A**) The three-dimensional (3D) structural model of PhcrTx2 (obtained by RaptorX) includes three antiparallel β-strands (residues 14–18 DKWIF, 30–34 DRCFM, and 37–42 GSVCCY); (**B**) Prediction of the ligand-binding residues of PhcrTx2 (the structural model is rotated on the *x*-axis, with respect to the (**A**) by CLIP-4D, using structural and evolutionary information from the multiple sequence alignment; (**C**) Surface electrostatic potential representation of PhcrTx2. The surface is colored according to the electrostatic potential: negative regions (in red), positive regions (in blue), and neutral regions (in gray). The orientation of the surface electrostatic potentials is the same as that in the ribbon representation (**A**). We also provided a color intensity scale to better represent the electrostatic potential. This figure was prepared with the PyMOL Molecular Graphics System, Schrödinger, LLC.

Putative 3D models of PhcrTx2 were obtained by using the protein structure prediction servers Swiss-Model, Phyre2, I-Tasser, LOMETS, and RaptorX. The proposed models were evaluated according to quality values and by the similarity to their corresponding template (Supplementary Table S3). The PhcrTx2 3D-model generated by RaptorX, which is based on the BDS-I structure (PDB code: 1bds), showed slightly superior quality values compared to the average of the others. Most of the sequence regions that were predicted to contain β-strands by RaptorX matched those predicted by JNET, PSSPRED, and PSIPRED (Figure 5).The 3D model includes three antiparallel β-strands (residues 14–18 DKWIF, 30–34 DRCFM, and 37–42 GSVCCY), a loop that connects the first and second strand, a turn between the second and third strand, and three disulfide bridges, as previously explained (Figure 6A).

According to the evolutionary information encoded within the multiple sequence alignment, in addition to Cys residues, several other highly conserved residues, such as Gly8, Gly13, Trp16, Pro23, Gly27, Tyr28, and Tyr42 should play structural/functional roles. It is expected that residues Gly and Pro contribute to the peptide conformation (structural role). Trp and Tyr residues may have a structural role, but also may have a functional role by promoting the interaction with other molecules such as ion channels and receptors. For the prediction of the structural or ligand-binding role of PhcrTx2 amino acid residues, we used CLIPS-4D. This classifier predicts a role in catalysis, ligand-binding, or protein stability in a mutually exclusively manner for each residue-position, which is assigned a P-value that enables the statistical assessment and the selection of predictions with similar quality. Prediction requires as input a multiple sequence alignment and a 3D structure of a protein in PDB format, and it exploits seven sequence-based and two structure-based features. Regarding the prediction of ligand-binding sites, the 3D structure is used to deduce the local environment of each residue; CLIPS-4D does not use the position of a ligand [45]. According to the classifier CLIPS-4D, all of the highly conserved residues mentioned above, and few other less conserved residues were predicted to have a structural (pSTRUCT > pLIG) or ligand-binding role (pLIG > pSTRUCT). More specifically, all of the Cys residues (which are engaged in disulfide bridges)—Gly8, Gly13, Trp16, Pro23, Gly27, Tyr28, and Gly37—should contribute to shaping the peptide structure. On the other hand, Tyr26, Asn29, and Tyr42 were predicted to have functional roles as ligand-binding residues (Figure 6B).

## 3. Discussion

Up to date, more than 30 crab-paralyzing toxins have been isolated from sea anemones. Many of these peptides have been classified as voltage-gated sodium channel toxins, whereas few other crab-paralyzing peptides act on different targets, such as voltage-gated potassium channels. On the other hand, other paralyzing toxins seem to belong to new classes of toxins [21,22]. In this work, we found a diversity of paralyzing toxins from the sea anemone *P. crucifer* by using a crab toxicity assay for biological activity screening during the purification process. Sixteen chromatographic fractions showing crab-paralyzing activity were isolated from the whole-body extract of *P. crucifer*. A new peptide toxin (PhcrTx2) was discovered from one of the crab-paralyzing fractions (Fraction 5 Figure 2A), which was selected for containing a relatively large amount of peptide, which is suitable for further chemical and pharmacological experiments. Also, fraction 5 was not lethal to crab, and the envenomation symptoms showed longer onset times, and were less severe than those provoked by sodium channel toxins (which are lethal to crabs), the most studied group of crab-paralyzing toxins from sea anemones. These facts led us to sequence PhcrTx2, and to investigate its pharmacology in an attempt to find new molecular targets of sea anemone toxins. This study represents the first report on paralyzing toxins from a sea anemone belonging to the family Phymanthidae. A previous report showed the isolation and characterization of the first ICK toxin (PhcrTx1) found in sea anemone, which is an ASIC inhibitor, and showed no paralyzing effects on crabs [6]. Although a similar chromatographic protocol was used for isolating both toxins (PhcrTx1 and PhcrTx2), their chromatographic behaviors are so contrasting that their purification strategy (regarding the selection of the active fraction to be purified) completely diverged after the cation-exchange chromatographic step (Figure 1B). Nonetheless, it is worthy to mention that both toxins also have different retention times in reversed-phase chromatography (Figure 2F), indicating that PhcrTx2 is more hydrophobic than PhcrTx1. On the other hand, PhcrTx1 is a much more basic peptide (pI 10.89 > 7.61) than PhcrTx2.

### 3.1. P. crucifer Produces a Diversity of Crab-Paralyzing Toxins

Besides PhcrTx2, *P. crucifer* produces several toxins with crab-paralyzing activity. In general, two types of envenomation signs were observed. First, fractions 1–4 and 6 provoked a lethal paralysis of barely perceptible signs, which were clearly different from the tetanic reactions. Second, fractions 5 and 7–16 induced tetanic reactions resembling, to some degree, the effects provoked by sodium channel toxins, although with different onset times and severity. Additionally, considering the basic/acidic properties, molecular masses, and hydrophobicities of these toxic fractions, the results suggest that *P. crucifer* produces five groups of crab-paralyzing toxins (Table 1, Supplementary Table S1). Further experiments should be performed to reveal the molecular targets of these toxins, as well as study the interactions between them.

For more than 40 years, the crab bioassay [17,18] has been widely used for the discovery of sea anemone toxins that have been mostly classified as sodium channels toxins. Interestingly, other types of peptide toxins, such as Am I and II from *Antheopsis maculata* [21], Gigantoxin I from *Stichodactyla gigantea* [22], and SHTX I and II from *Stichodacyla haddoni* [20], have been discovered through the careful observation of signs in crabs. The variety of paralyzing toxins found in our study supports the importance of such observations to widen the application of the crab bioassay in the discovery of new toxins and families of toxins in sea anemones.

### 3.2. PhcrTx2 Is a New Defensin-Like Toxin

The computational analysis (Figure 5 and Figure 6A) indicates that PhcrTx2 is a new member of the β-defensin-fold family of polypeptides, according to several distinctive features previously described [44]. More specifically, PhcrTx2 should be a new member of the “Defensin 4” family (Pfam accession: PF07936), which comprises several β-defensin-fold peptide toxins from sea anemones.

Three ligand-binding residues (Tyr26, Asn29, and Tyr42) were predicted for PhcrTx2 according to the evolutionary information encoded within the multiple sequence alignment and the PhcrTx2 three-dimensional model. Although this proposal is based on theoretical considerations, this information could be used in principle as a reference for structure–function analyses of recombinant or chemically synthesized variants, to determine the functional sites of this toxin. Also, given that highly conserved residues in a multiple sequence alignment can be of biological significance, new structural or functional residues from toxins similar to PhcrTx2 could be identified from the multiple sequence alignment. In addition to Cys residues, other conserved residues such as Gly9, Gly14, Trp17, Pro25, Gly29, Tyr30, Thr31, and Tyr44 (according to the Multiple Sequence Analysis numbering in Figure 5) may play a structural/functional role in their interaction with molecular targets, such as ion channels and receptors. To date, few experimental studies have been reported regarding the functional residues of these toxins; most of these studies have been focused on APETx2, [5,46,47,48].

### 3.3. PhcrTx2 Is a Crab-Paralyzing Toxin Exhibiting a Low Potency Activity on Ion Channels

Most known crab-paralyzing toxins from sea anemones have been classified into the groups of Na_v_ toxins. A minor group has been included among the K_v_ toxins, whereas other crab-paralyzing toxins remain unclassified because of the lack of pharmacological studies to characterize their mode of action, and the absence of sequence similarity to other groups of sea anemone toxins [49]. Although PhcrTx2 was paralyzing on *U. thayeri* crabs with an ED_50_ = 707 µg/kg, it induced no severe envenomation signs or death, in contrast to the effects currently provoked by known sodium channel toxins from sea anemones [27], which are potently lethal to crabs. The toxicity effects of this toxin in crabs resemble the ones reported for Am II for *Antheopsis maculata* [25], whose targets remain unknown. Only a small inhibition of Na_v_ current was detected in DRG neurons by the toxin action. No activity of PhcrTx2 at micromolar concentrations was detected in cloned human ion channels nor in cloned insect Na_v_ channel DmNa_v_1 (data not shown), which is considered a very sensitive model for the evaluation of crustacean toxins [50].

PhcrTx2 inhibited the K_v_ current with low potency and efficacy in rat DRG. On the other hand, PhcrTx2 had no influence on a large variety of cloned human K_v_ channels, even at a concentration of 5 µM. This included K_v_3.1, which is inhibited by BDS-I and BDS-II [34,35]. Whether the activity detected on native K_v_ currents could be linked to the paralyzing activity of PhcrTx2 is still unknown. 

Sea anemones can prey on vertebrates such as fish and small invertebrates, which are mainly crabs and molluscs. Interestingly, the pharmacology of snail neurons is similar to the pharmacology of insect and crustacean central neurons [51]. Then, the use of snail neurons as a model for the characterization of toxins provides an adequate system, since their ion channels could be targeted by these toxins under natural conditions. The use of DRG neurons, despite they are very different taxonomically, represents an important step for comparison with previous results obtained from other sea anemone toxins [52,53,54]. Furthermore, depending on the route of administration, when tested in mice, some anemone toxins may have LD_50_ levels that are several orders of magnitude below those obtained when tested on crabs. For example, the RpI toxin (*from Radianthus paumotensis*) has an LD_50_ of 36 μg/kg when tested on crabs, whereas its LD_50_ is only 1.5 μg/kg when injected intracisternally into mice [26]; on the other hand, Av2 (from *Anemonia viridis*) is highly active in both crabs and mice [55]. Thus, DRG neurons represent a suitable model for studying the pharmacological action of anemone toxins.

According to the present results, PhcrTx2 inhibited glutamate currents in snail neurons. However, no specific subtype of glutamate receptors was determined, and it will be of great interest to study the toxin’s specificity in the future using different models. Although the PhcrTx2 inhibitory activity on glutamate-gated currents exhibited low potency and efficacy, this study supports the idea of the existence of glutamate-gated ion channel toxins in these organisms, as previous studies have indicated through the evaluation of a semi-pure chromatographic fraction and a sea anemone crude extract [56,57]. Thus, our results represent a step forward toward the finding of a glutamate-gated ion channel toxin in sea anemone. It would be interesting to test other structurally related toxins on glutamate-gated ion channels, especially Am-2 [21], which shares high sequence similarity with PhcrTx2 and might have common targets.

On the other hand, PhcrTx2 also inhibited voltage-gated K^+^ channels in DRG neurons with low potency and efficacy. DRG neurons express a wide variety of K_v_ channels, including those with rapid inactivation (IKA) and delayed rectifier (IKDR) channels. In DRG neurons, we identified a transient component that is presumably related to IKA, and a sustained component related to the IKDR contribution. The toxin seems to affect both current components without a preferential inhibitory activity on either of them.

The inhibition of PhcrTx2 on glutamate-gated currents in snail neurons, and also on voltage-activated K^+^ currents in DRG neurons, seems to be of about similar potency and slightly different in efficacy. PhcrTx2 also acted on voltage-dependent Na^+^-channels of DRG neurons but with lower efficacy, in comparison with its effect on K^+^ channels. According to our results, the toxin shows a pleomorphic action across different ion channels as it acts on voltage-dependent K^+^ channels, as well as on glutamate-activated channels, although with low potency and efficacy. These facts resemble previous results from other sea anemone toxins. PhcrTx1, the first sea anemone ICK peptide characterized from *Phymanthus crucifer*, constitutes a novel acid-sensing ion channel (ASIC) inhibitor, but it also inhibited the voltage-gated K^+^ currents in DRG neurons, although with lower efficacy and potency. Studies carried out with sea anemone toxins APETx1 and APETx2, which are from a different structural family produced by the species *Anthopleura elegantissima*, demonstrated their lack of target selectivity, even though these toxins were considered highly selective for some time [5,38]. APETx2 was initially considered as a specific reversible blocker of the peak component of the ASIC3 current (IC_50_ = 63 nM), with no effect on the sustained component of the current. This toxin also affects heteromeric channels that contain the ASIC3 subunit, except the ASIC2a + 3 current [38]. However, it has been shown that APETx2 has promiscuous properties, since it partially inhibits K_v_3.4 channels, and recognizes insect and mammalian Na_v_ channels inhibiting the sodium conductance without affecting the inactivation and shifting activation to a depolarizing direction [38,58]. Interestingly, a recent study has shown that APETx2 potentiates the ASIC1b current at concentrations that are 30-fold to 100-fold higher than the concentration inhibiting ASIC3 [59].

From a biological point of view, the simultaneous action of the toxin on different targets involved in the basic mechanism of the function of nervous system could be a successful strategy for survival. However, given the low potency and efficacy of PhcrTx2 action on the evaluated ion channels, we cannot discard the possibility that the main molecular target of this toxin remains unknown. Further studies should be designed in the future to evaluate PhcrTx2 on a larger variety of receptors and ion channels.

## 4. Conclusions

In the present study, we explored the diversity of crab-paralyzing toxins from the aqueous extract of *P. crucifer*. Our study represents the first report of paralyzing toxins in the species *P. crucifer* and its family Phymanthidae. The careful observation of the paralysis signs, together with the biochemical properties of the toxic chromatographic fractions, allowed predicting five groups of paralyzing toxins in *P. crucifer*. Further experiments will be crucial for revealing the structure and pharmacological targets of these molecules. PhcrTx2, a new paralyzing peptide, was isolated and characterized. This toxin represents a new member of the defensin-fold family of peptides, and shares sequence similarity with type 3 potassium channel toxins from sea anemones. However, its weak activity on ion channels commonly targeted by other members of this structural group aims at the existence of other molecular targets that should contribute to revealing the origin of the toxicity of PhcrTx2.

## 5. Materials and Methods

### 5.1. Chromatographic Separation of the Toxic Compounds

A five gram-sample of a *P.* *crucifer* freeze-dried whole homogenate prepared in a previous work [6] was mixed with 100 mL of 0.1 M ammonium acetate (p.a, Merck, Darmstadt, Germany), stirred for 30 min, and centrifuged at 4000 g for one hour at 4 °C. The supernatant (350 mg/90 mL) was applied to a Sephadex G-50 M column (Pharmacia, Stockholm, Sweden) of dimensions 5 cm × 93 cm. The separation was done at a flow rate of 2 mL/min using 0.1 M ammonium acetate (pH 6.96) as eluent. Fractions of 20 mL each were collected and manually read at 280 nm in a UV-1201 spectrophotometer (Shimadzu, Kyoto, Japan).

The biologically active sample from gel filtration was applied to a Fractogel EMD SO_3_^−^ 650 M (Merck, Darmstadt, Germany) cation-exchange column of dimensions 1.8 × 5 cm. The non-retained fraction was applied to a Fractogel EMD DEAE 650 M (Merck, Darmstadt, Germany) anion-exchange column of dimensions 1.8 × 5 cm. Both ion-exchange steps were performed separately under the following conditions: a 400-mL (31 column volumes) gradient of ammonium acetate was run at a flow rate of 1 mL/min, from 0.01 M (pH 6.90) to 1 M (pH 7.20), using a gradient mixer GM-1 (Pharmacia, Stockholm, Sweden). Eighty fractions of 5 mL each were collected and manually read at 280 nm in a spectrophotometer.

The biologically active samples from ion-exchange chromatography were submitted to reversed-phase chromatography on a Hypersil H5 ODS column (Unicam, Cambridge, UK) of dimensions 4.6 mm × 250 mm, which was previously equilibrated with solvent A, 0.1% trifluoroacetic acid (HPLC grade, AppliChem, Darmstadt, Germany). Elution was carried out at a flow rate of 0.8 mL/min by using an ascending linear gradient of solvent B, 0.05% TFA in acetonitrile, (0–80% B in 80 min). The peptide was repurified in a Discovery RPC18 HPLC column (Supelco, Bellefonte, PA, USA) of dimensions 4.6 mm × 250 mm, using the gradient 10–20% B in 5 min, and then 20–30% in 50 min, at the flow rate of 1 mL/min. Eluting compounds were detected at 214 nm.

The protein content of the aqueous extract and chromatographic fractions was estimated with a Bicinchoninic acidprotein assay kit (AppliChem, Germany) [60].

### 5.2. Biological Assays

#### 5.2.1. Crab Toxicity Assay

The chromatographic fractions were assayed on male shore crabs *Uca thayeri* weighing 2–4 g, according to a crab bioassay widely used for the detection of paralyzing effects induced by sea anemone toxins [17,22]. The samples were injected (10 µL/g crab weight) into the base of the third walking leg, at a concentration of 0.2 mg/mL (corresponding to the dose of 2000 µg/kg) for toxicity screening purposes. Three crabs were used per sample, and the paralyzing activity was considered positive at the inability of all of the crabs (placed upward facing) to right themselves within two hours after toxin administration. After the observation of paralyzing effects, the crabs were killed. For the estimation of the ED_50_ value, groups of six crabs were used per dose. A non-linear fit of log (dose) versus normalized response (variable slope) was performed using GraphPad Prism version 7.03 for Windows (GraphPad Software, La Jolla, CA, USA, www.graphpad.com).

#### 5.2.2. Patch Clamp Experiments on Neuronal Ion Channels

The effects of PhcrTx2 were studied in isolated mammalian and snail neurons using the whole cell patch-clamp technique. Animal care and procedures were in accordance with the National Institutes of Health Guide for the Care and Use of Laboratory Animals. All efforts were made to minimize animal suffering. The number of animals used for this work was kept to the minimum necessary for a meaningful interpretation of the data.

##### Cell Culture of Rat Dorsal Root Ganglion (DRG) Neurons and Electrophysiological Recordings

DRG neurons from Wistar rats (P5 to P9) were isolated and cultured according to the previously described procedure [52,61]. The rats were anesthetized and killed with an overdose of sevofluorane. The dorsal root ganglia were isolated from the vertebral column and incubated (30 min at 37 °C) in Leibovitz L15 medium (L15) (Invitrogen, Carlsbad, CA, USA) containing 1.25 mg/mL trypsin and 1.25 mg/mL collagenase (both from Sigma-Aldrich, St. Louis, MO, USA). The ganglia were then washed three times with sterile L15, and the cells were mechanically dissociated and plated on glass coverslips (Corning, Corning, NY, USA) pretreated with poly-D-lysine (Sigma-Aldrich), and placed onto 35-mm culture dishes (Corning). Neurons were incubated 4 h to 8 h in a humidified atmosphere (95% air, 5% CO_2_, at 37 °C) in a CO_2_ water-jacketed incubator (Nuaire, Plymouth, MN, USA). The medium contained L15, with 15.7 mM NaHCO_3_ (Merck, Naucalpan, Mexico), 10% fetal bovine serum, 2.5 μg/mL fungizone (both from Invitrogen), 100 U/mL penicillin (Lakeside, Toluca, Mexico), and 15.8 mM HEPES (Sigma-Aldrich).

The coverslip with attached neurons was transferred to a 500-mL perfusion chamber on the stage of an inverted phase contrast microscope (Nikon Diaphot, Tokyo, Japan). To study the effect of the toxin on voltage-activated Na^+^ and K^+^ currents, the cells were bathed at a rate of about 100 μL/min, with the external solutions shown in Supplementary Table S4.

A pair of glass capillaries placed approximately 40 μm above the cell continuously microperfused (20 μL/min) external solution or external solution plus toxin. Patch pipettes were pulled from borosilicate glass capillaries (TW120-3; WPI, Sarasota, FL, USA) using a Flaming–Brown electrode puller (P80/PC; Sutter Instruments, San Rafael, CA, USA). They typically had a resistance between 1–2.5 MΩ when filled with the internal solution (Supplementary Table S4). The whole-cell patch clamp technique was used to record ionic currents with an Axopatch-1D amplifier (Molecular Devices, Sunnyvale, CA, USA). Command pulse generation and data sampling were controlled by the PClamp 8.0 software (Molecular Devices) using a 16-bit data acquisition system (Digidata 1320A, Molecular Devices). Signals were low-pass filtered at 5 kHz or 10 kHz, and digitized at 20 kHz. Capacitance and series resistance (80%) were electronically compensated. During the experiment, seal and series resistance were monitored to guarantee stable recording conditions. The K^+^ currents were elicited by a single-step voltage protocol from −100 mV (V_hold_) to 0 mV during 800 ms every 8 s. Na^+^ currents were elicited by pulses from −100 mV (V_hold_) to −10 mV during 40 ms every 8 s. It has been reported that activation and steady-state inactivation curves shift over time in whole-cell patch clamp experiments [62]. The recordings were initiated 10–15 min after the whole-cell configuration was established, in order to minimize the effects of time-dependent shifts.

To study the effects on voltage-gated Na^+^ and K^+^ currents, the toxin was ejected under pressure using a microinjector (Baby Bee; Bass, West Lafayette, IN, USA) from a micropipette positioned in the vicinity of the cell under recording, which was perfused with the toxin until the steady state of the effect was reached.

The concentration-response curves were fitted to the Hill equation: Y = Y_max_ × x_n_/(k_n_ + x_n_), where Y is the effect of the substance under study, Y_max_ is the maximum effect, x is the concentration of the toxin, k is the concentration that produces half the maximum effect, and n is the Hill coefficient. To define the statistical significance, the control recordings were compared with those obtained after toxin perfusion by using a paired Student’s *t*-test; a *p* ≤ 0.05 was considered as significant. Unless otherwise stated, all of the numerical data are presented as the mean ± SEM.

##### Voltage Clamp Experiments on Isolated Snail Neurons

Experiments to study glutamate-activated currents were performed in isolated neurons of the land snail *Helix aspersa*. The subesophageal mass of the snail was dissected, and put into the Ringer solution for snails (Supplementary Table S4) containing trypsin (2 mg/mL for 30 min, at 37 °C). After enzymatic treatment, the preparation was washed with normal solution for snails, and the neurons were mechanically dissociated, plated on glass coverslips (Corning) pretreated with poly-D-lysine (Sigma-Aldrich), and placed onto 35-mm culture dishes (Corning). Neurons were maintained at room temperature (23–25 °C) for at least 30 min before using, in order to allow the isolated cells to adhere to the coverslips. The whole-cell recording was carried out using the same experimental conditions that were described in Section 5.2.2.

Glutamate currents were elicited by a fast glutamate application (about 40 ms) by shifting one of the three outlets of a fast change perfusion system (SF-77B, Warner Inst., Hamden, CT, USA) while keeping the cell at a holding potential (V_h_) of −100 mV. The interval between glutamate applications was one minute, to guarantee that the current completely recovered from desensitization. The pH of glutamate solution was checked before each experiment to avoid the potential activation of proton-gated currents. At the beginning of this study, experiments were designed to compare the effect of the toxin using two application protocols. No differences were observed in the toxin’s action (10 μM) when the toxin was preapplied 10 s before and during glutamate ejection (sustained application), or when it was coapplied with glutamate. In the case of sustained application, we also did not observe significant differences in the amplitude of the current between the first pulse of glutamate and a subsequent one applied one minute later, in whose interval the perfusion of the toxin was not suspended. In view of these results, we decided to carry out the rest of the experiments using the co-application protocol.

The solutions used in the experiments are depicted in Supplementary Table S4. The concentration-response curves were fitted to the Hill equation following the procedure described in Section 5.2.2.

#### 5.2.3. Expression of Voltage-Gated Ion Channels in *Xenopus laevis* Oocytes and Electrophysiological Recordings

For the expression of the voltage-gated potassium channels (K_v_1.1, K_v_1.2, K_v_1.3, K_v_1.4, K_v_1.6, *Shaker* IR, K_v_2.1, K_v_3.1, K_v_4.2, K_v_10.1) and voltage-gated sodium channels (Na_v_1.4, Na_v_1.5, Na_v_1.6, Na_v_1.8 and DmNa_v_1) in *Xenopus laevis* oocytes, the linearized plasmids were transcribed using the T7 or SP6 mMESSAGE-mMACHINE transcription kit (Ambion, Foster City, CA, USA). The harvesting of stage V-VI oocytes from an anesthetized female *Xenopus laevis* frog was previously described [63]. Oocytes were injected with 50 nL of cRNA at a concentration of 1 ng/nL using a microinjector (Drummond Scientific, Broomall, PA, USA). The oocytes were incubated in ND96 solution (Supplementary Table S4), which was supplemented with 50 mg/L gentamycin sulfate. The use of the frogs was in accordance with the license number LA1210239.

Two-electrode voltage-clamp recordings were performed at room temperature (18–22 °C) using a Geneclamp 500 amplifier (Molecular Devices, USA) controlled by a pClamp data acquisition system (Axon Instruments, Union City, CA, USA). Whole-cell currents from oocytes were recorded 1–4 days after injection. Bath solution composition was ND96 (Supplementary Table S4). Voltage and current electrodes were filled with 3 M KCl. Resistances of both electrodes were kept between 0.7–1.5 MΩ. The elicited currents were filtered at 1 kHz, and sampled at 500 Hz using a four-pole low-pass Bessel filter. Leak subtraction was performed using a −P/4 protocol. K_V_1.1–K_V_1.6 and Shaker IR currents were evoked by 500 ms depolarizations to 0 mV followed by a 500 ms pulse to −50 mV, from a holding potential of −90 mV. K_V_2.1, K_V_3.1, and K_V_4.2, currents were elicited by 500 ms pulses to +20 mV from a holding potential of −90 mV. 

### 5.3. Mass Spectrometry and N-Terminal Sequencing

The molecular mass analysis of the RP-HPLC fractions was performed with a Voyager-DE Pro matrix-assisted laser desorption/ionization time-of-flight mass spectrometry (MALDI–TOF–MS) device (PerSeptive Biosystems, Framingham, MA, USA). The matrix solution was prepared with α-cyano-4-hydroxycinnamic acid dissolved at 5 mg/mL in mass buffer (0.1% TFA in 1:1 acetonitrile/water solution). One microliter of the sample solution and matrix solution were mixed on a 100-well stainless steel MALDI plate. Measurements were performed in linear mode. Positive ions were accelerated at 20 kV, and up to 100 laser shots were automatically accumulated per sample position. Voyager RP BioSpectrometry Workstation version 3.07.1 (PerSeptive Biosystems, USA) was used as the control software.

The peptide sample was dissolved in 10% acetonitrile in water (*v*/*v*), and spotted on a glass fiber disk (Wako, Japan), which was pretreated with Sequa-brene (Sigma, USA). Sequences were determined by automated Edman degradation using a ShimadzuPPSQ-30 protein sequencer (Tokyo, Japan) according to the manufacturer’s instructions.

### 5.4. Computational Analyses

The amino acid sequence of PhcrTx2 was submitted to different webservers for an in silico characterization. The software GPMAW 10.2 (http://www.welcome.to/gpmaw) [64] was used for the theoretical calculations of the isoelectric point and average molecular mass. Sequences and the three-dimensional (3D) structures of similar peptides were retrieved from the UniProt/Swiss-Prot and the Protein Data Bank (PDB) databases, respectively. PSI-BLAST (NCBI BLAST 2.0) (http://www.ebi.ac.uk/Tools/sss/psiblast/) [65] and Pfam 31.0 (http://pfam.xfam.org/) [40] were used to identify PhcrTx2 homologues based on sequence similarity and domain architecture, respectively. Sequence alignments with a bit-score greater than 100 and an E-value of less than 0.001 were considered significant. The sequences mined from Pfam were selected in order of descending score, according to pairwise sequence alignments with PhcrTx2 using the EMBOSS water tool 6.6.0.0 (http://www.ebi.ac.uk/Tools/psa/emboss_water/) [66]. MAFFT v7 (using L-INS-i as iterative refinement method, http://mafft.cbrc.jp/alignment/server/) [67] and Jalview 2.8.2 (http://www.jalview.org/) [68] were used for multiple sequence alignment. Secondary structure prediction was performed with PSSpred v3 (https://zhanglab.ccmb.med.umich.edu/PSSpred) and PSIpred v3.3 (http://bioinf.cs.ucl.ac.uk/psipred/) [69]. The three-dimensional (3D) structure of PhcrTx2 was predicted by combining a comparative modeling strategy (i.e., SWISS-MODEL in the automated mode at http://swissmodel.expasy.org/workspace/index.php?func=modelling_simple1&userid= USERID&token=TOKEN [70]) with a template-free approach (i.e., Phyre2 v2.0 at http://www.sbg.bio.ic.ac.uk/phyre2 [71], I-Tasser v5.0 at https://zhanglab.ccmb.med.umich.edu/I-TASSER/ [72], LOMETS v4.0 at http://zhanglab.ccmb.med.umich.edu/LOMETS/ [73], and RaptorXat http://raptorx.uchicago.edu/ [74]). The predicted 3D models of PhcrTx2 were subjected to a series of tests for evaluating their internal consistency and reliability. Backbone conformation was evaluated by the inspection of the Psi/Phi Ramachandran plot obtained from PROCHECK analysis [75]. Packing quality of the 3D model was investigated by the calculation of the WHATCHECK *Z*-score value [76]. Lastly, sequence–structure compatibility was evaluated by VERIFY3D [77]. PROCHECK, WHATCHECK, and VERIFY3D were executed from the structure analysis and verification servers’ website at UCLA (https://services.mbi.ucla.edu/SAVES/). Also, we used Qmean *Z*-score (https://swissmodel.expasy.org/qmean/) for the absolute quality assessment of the peptide models [78]. CLIPS-4D (https://bioinf.ur.de/clips4d.php) [45] was used to distinguish structurally and functionally important residue-positions based on the multiple sequence alignment and 3D data. Swiss-PdbViewer 4.1.0 (http://www.expasy.org/spdbv/) [79] was used to visualize the PhcrTx2 3D model. The PyMOL Molecular Graphics System, Version 1.8 Schrödinger, LLC was used to visualize the electrostatic potential molecular surface of PhcrTx2.

## Figures and Tables

**Table 1:** Features overview of distinct groups of toxic reversed-phase chromatographic fractions, based on chromatographic behavior, molecular masses, and crab-paralyzing activity. Three crabs were used per sample for screening purposes. Only those samples provoking death or paralysis to all of the crabs were considered lethal or paralyzing, respectively, at 2000 μg/kg.

RP-HPLC Fraction Number	Features	Paralyzing Effects on Crab *Uca thayeri*
1–4 and 6	t_R_ = 25–30 min * 1986–5671 Da ** basic peptides ***	Progressive slowing down of legs movements until remaining motionless approximately after 30 min. Lethal.
5 and 7	t_R_ = 40–45 min 5294–5296 Da basic peptides	Moderate tetanic paralysis starting after 30 min from toxin administration. Partial recovery from paralysis followed by uncoordinated leg movements. Not lethal.
8	t_R_ = 51 min 3082 Da basic peptide	Severe tetanic paralysis after a few seconds, resembling the effects provoked by Na_v_ toxins [27]. Lethal.
9–15	t_R_ = 49–59 min 4573–9847 Da acidic peptides	Severe tetanic paralysis after a few seconds, resembling the effects provoked by Na_v_ toxins [27]. Lethal.
16	t_R_ = 50–55 min 19,758 Da acidic protein	Moderate tetanic paralysis 5 min after toxin administration. Not lethal.

* In the reversed-phase HPLC separation. ** Measured by MALDI-TOF-MS (see Supplementary Table S1). *** According to their retention in ion-exchange chromatography at pH 7: the basic peptides were retained in the cation exchanger, whereas the acidic proteins and peptides were retained in the anion exchanger.

## Supplementary Materials

The following are available online at http://www.mdpi.com/2072-6651/10/2/72/s1, Figure S1: Dose-response curve of the PhcrTx2 paralyzing activity on crabs, Figure S2: PhcrTx2 action on voltage-dependent Na^+^ and K^+^ currents in DRG neurons, Figure S3: Activity profile of PhcrTx2 on K_v_ and Na_v_ channels expressed in *Xenopus laevis* oocytes, Table S1: Molecular mass and retention time data of toxic reversed-phase chromatographic fractions, Table S2: Current amplitude PhcrTx2/control, Table S3: Model quality assessment and Table S4: Solutions used in the whole-cell patch/voltage clamp experiments.
