# Supplementary material for: PhcrTx2, a New Crab-Paralyzing Peptide Toxin from the Sea Anemone Phymanthus crucifer
Source: Toxins (Basel). 2018 Feb 7;10(2):72. doi: 10.3390/toxins10020072 (PMC5848173; doi:10.3390/toxins10020072)
Supplement: Supplementary file 1 [file toxins-10-00072-s001.pdf]

Supplementary Materials: PhcrTx2, a new crab-paralyzing peptide toxin from the sea anemone *Phymanthus crucifer*

Armando Alexei Rodríguez, Anoland Garateix, Emilio Salceda, Steve Peigneur, André Junqueira Zaharenko, Tirso Pons, Yúlica Santos, Roberto Arreguín, Ludger Ständker, Wolf-Georg Forssmann, Jan Tytgat, Rosario Vega and Enrique Soto

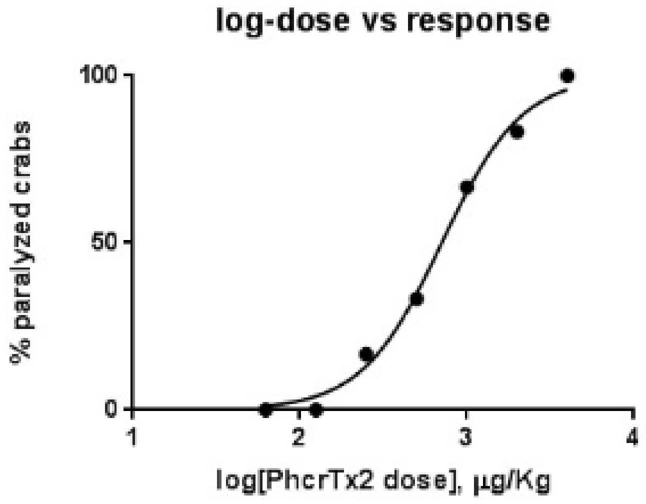

Figure S1. Dose-response curve of the PhcrTx2 paralyzing activity on crabs. A nonlinear fit of log (PhcrTx2 dose, μg/kg) vs normalized response (from 0 to 100% paralyzed crabs) was performed using GraphPad Prism 7.03. The calculated ED50 value was 707μg/kg. The values (X ug/kg: Y %) were 62.5 ug/kg: 0%, 125 ug/kg: 0%, 250 ug/kg: 16.7 %, 500 ug/kg: 33.3%, 1000 ug/kg: 66.7%, 2000 ug/kg: 83.3% and 4000 ug/kg: 100%

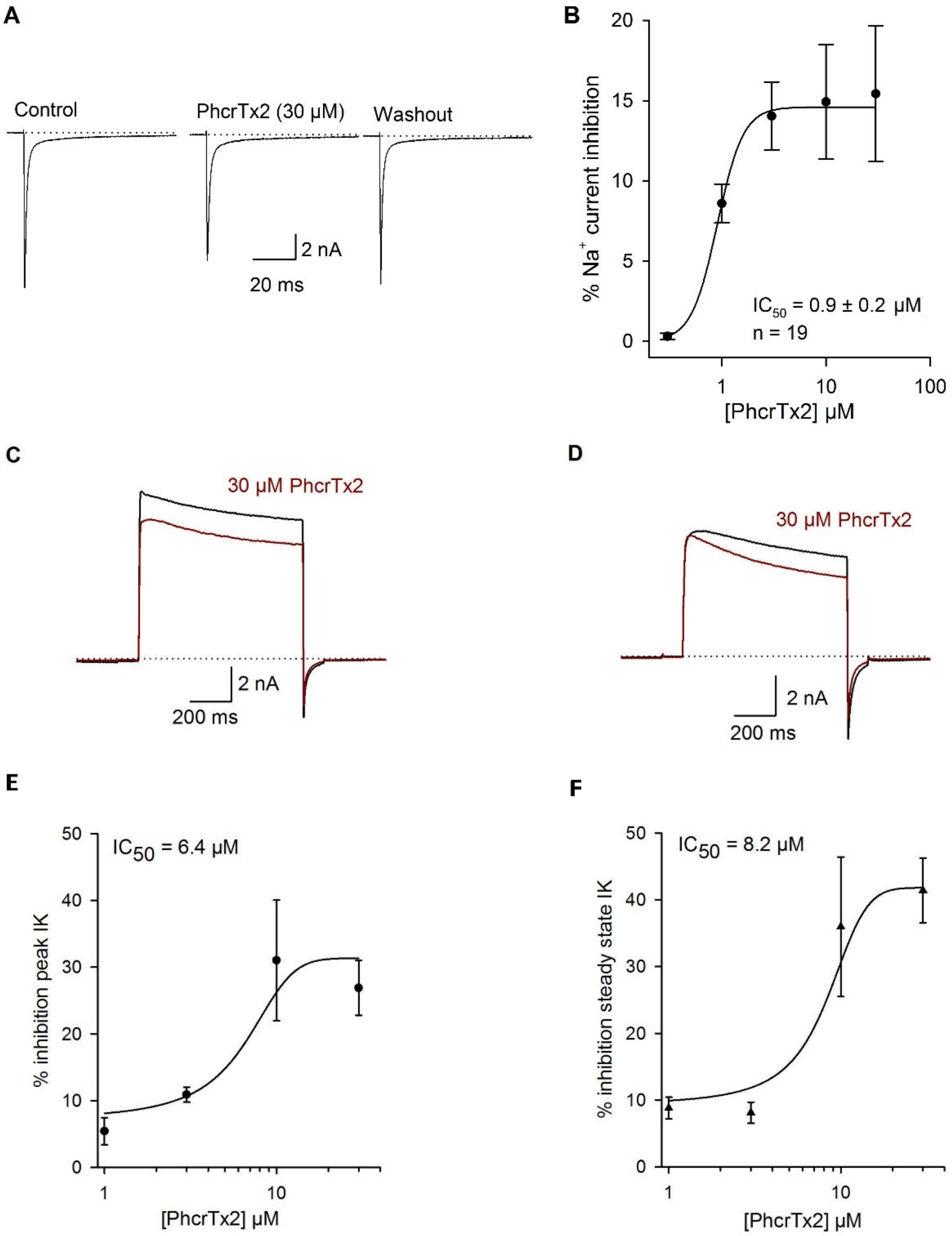

Figure S2. PhcrTx2 action on voltage-dependent Na+ (A and B) and K+ currents (C to F) in DRG neurons. (**A**) Typical experiment showing isolated Na+ currents in DRG neurons under control condition, after the application of 30 μM PhcrTx2 and after washout of the toxin. (**B**) Concentration-response relationship for the PhcrTx2 inhibitory action on the peak Na+ current. Data were fitted to a dose-response curve, with an IC_{50} = 0.9 ± 0.2 μM. C and D) K+ current traces (different cells for **C** and **D**) under control condition and in 30 μM PhcrTx2 presence. The toxin produced inhibition of the K+ current peak amplitude and the steady state current components. E) Concentration-response curve of the PhcrTx2 inhibition of the peak current (IC_{50}= 6.4 ± 0.2 μM). F) Concentration-response curve for the inhibition produced by PhcrTx2 in the steady-state current component (IC_{50} = 8.2 ± 0.7 μM). The toxin inhibited both components of the K+ current in a concentration-dependent manner (n = 31).

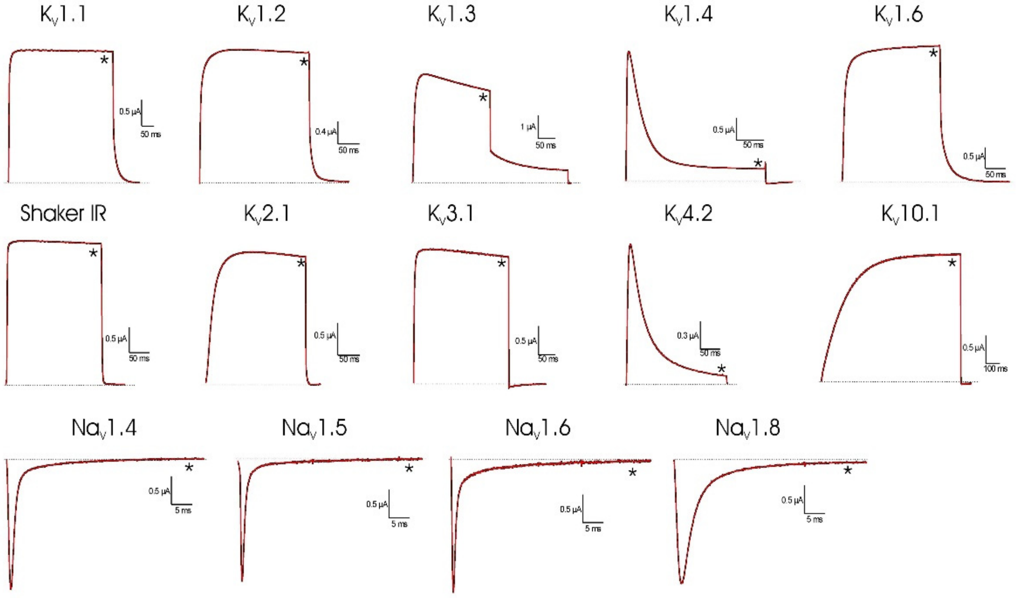

Figure S3. Activity profile of PhcrTx2 on K_V and Na_V channels expressed in *Xenopus laevis* oocytes. Representative whole-cell current traces in control and toxin conditions are shown. The dotted line indicates the zero-current level. The asterisk marks steady-state current traces after application of 5 µM peptide. No significant activity of PhcrTx2 was found on any of these ion channels. Panels show superimposed representative current traces for each channel type. The dotted line indicates zero current level. In black: the current trace in control conditions; in red: selected current trace after toxin application. Each experiment was performed at least three times ($n \geq 3$). Data are presented as mean ± S.E. Kv1.1-Kv1.6 and Shaker IR currents were evoked by 500 ms depolarizations to 0 mV followed by a 500 ms pulse to -50 mV, from a holding potential of -90 mV. Kv2.1, Kv3.1, and Kv4.2, currents were elicited by 500 ms pulses to +20 mV from a holding potential of -90 mV. Nav1.4-Nav1.6 and Nav1.8 traces were evoked by 100 ms depolarizations to Vmax (the voltage corresponding to maximal sodium current in control conditions).

Table S1. Molecular mass and retention time data of the 16 toxic reversed-phase chromatographic fractions. From left to right: Retention times of the reversed-phase chromatographic fractions, corresponding ion-exchange chromatographic fraction of origin (I to IV), molecular masses of the components found in every reversed-phase C18 chromatographic fraction, and the results of the crab-paralyzing activity screening, shown as "+" when the chromatographic fraction was only paralyzing, and "++" when it was lethal, at the dose of 2000 µg/Kg. Three crabs were used per sample for screening purposes. Only those samples provoking death or paralysis to all crab were considered as lethal or paralyzing, respectively, at 2000 µg/kg. The dividing line in the table separates basic peptides (eluted from cation-exchange chromatography at neutral pH) from acidic peptides (eluted from anion-exchange chromatography at neutral pH). Graphs in the second page show the peptide maps of the toxic reversed-phase chromatographic fractions, comprising molecular mass vs. retention time in reversed-phase chromatography. These maps allowed distinguishing five groups of toxins, which are indicated by circles.

RP fraction number	Retention time (min)	Ion-exchange fraction	Molecular mass (Da)	Crab-paralyzing activity
1	26.0	I	3107	++
	26.0		2076	
	26.0		2396	

	26.0		3048	
	26.0		3362	
	26.0		1986	
2	27.2	I	3081	++
	27.2		3270	
3	29.2	I	3110	++
	29.2		3060	
	29.2		3126	
	29.2		1987	
4	30.0	I	3074	++
	30.0		5671	
5	40.7	I	5296	+
6	30.0	II	2705	+
7	45.0	II	5294	
8	51.0	II	3082	
9	49.2	III	6745	++
10	53.0	III	6516	++
	53.0		4968	
	53.0		6940	
11	53.7	III	4573	++
12	54.2	III	6545	++
	54.2		7041	
	54.2		9847	
13	57.0	III	6655	++
14	57.5	III	6652	++
15	58.7	III	5028	+
16	58.5	IV	19758	+

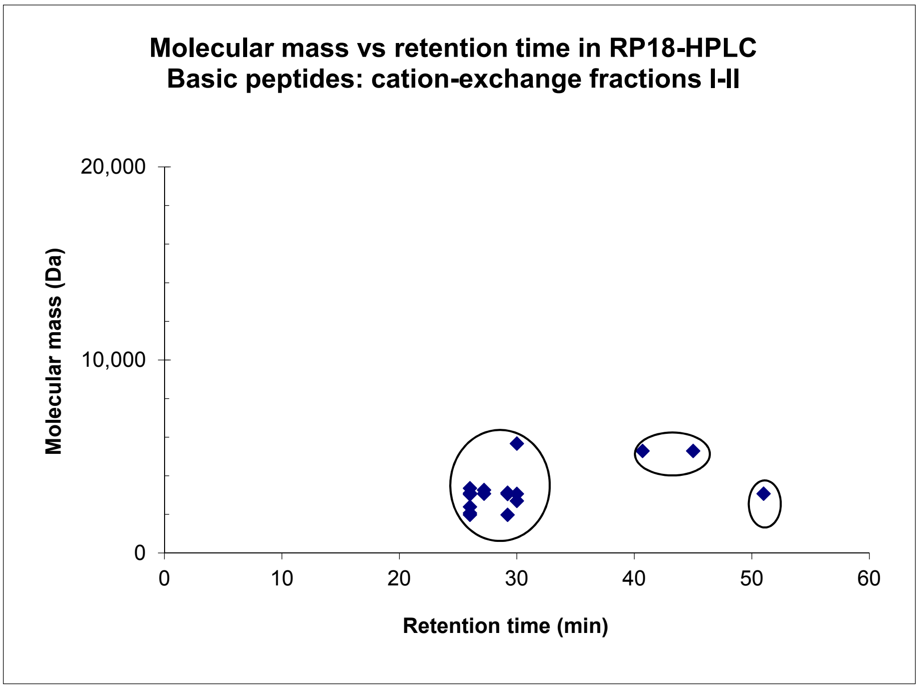

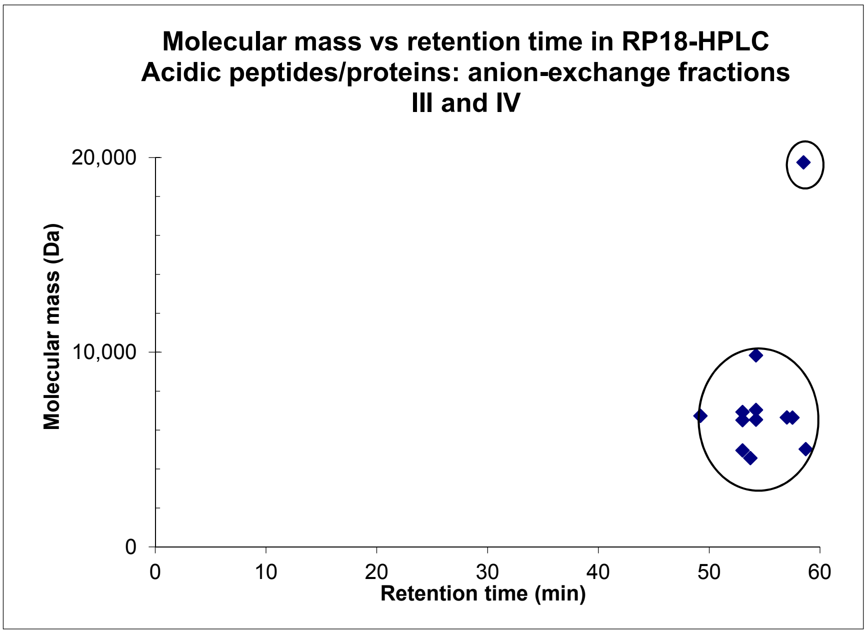

Table S2. Ratio of current amplitude in the presence of PhcrTx2 to control current amplitude.

Ion channel	Itoxin/Icontrol	nuber of cells
Kv1.1	0,98 ± 0,02	4
Kv1.2	0,99 ± 0,02	4
Kv1.3	0,97 ± 0,01	5
Kv1.4	0,99 ± 0,02	6
Kv1.6	0,97 ± 0,03	4
Kv2.1	1,01 ± 0,03	5
Kv3.1	0,98 ± 0,03	5
Kv4.2	1,00 ± 0,02	5
Kv10.1	0,96 ± 0,03	6
Shaker IR	1,02 ± 0,03	4
Nav1.4	0,99 ± 0,02	3
Nav1.5	0,99 ± 0,03	4
Nav1.6	0,98 ± 0,01	5
Nav1.8	0,97 ± 0,02	4

Table S3. Model quality assessment. Different structural models of the toxin PhcrTx2, obtained by homology modelling in SWISS-MODEL (automated mode), and by a fold recognition approach in Phyre2, I-Tasser, LOMETS and RaptorX, were evaluated by comparing several parameters calculated in PROCHECK (most favored regions, additional allowed regions, generally allowed regions and disallowed regions), WHATCHECK (2nd generation packing quality), VERIFY3D (3D-1D score: in all models > 80% residues scored above 0.2, which is the minimal correct value) with values obtained from their respective templates, BDS-I (UniProtKB P11494, PDB 1bds) and APETx1 (UniProtKB P61541, PDB 1wqk). The absolute quality of every structural model was evaluated by Qmean. N/A: not applicable to templates but only to structural models.

	3-D structural models										Templates	
**Characteristic**	**Swiss-model 1 (1BDS)**	**Swiss-model 2 (1BDS)**	**Swiss-model 3 (1BDS)**	**Phyre2-model 1 c1bds**	**Phyre2-model 2 d1bds**	**Lomets-model 1 (1wqk)**	**Lomets-model 2 (1BDS)**	**Lomets-model 3 (1BDS)**	**I-Tasser (1wqk)**	**RaptorX (1BDS)**	**1BDS (BDS-I)**	**1wqk (APETx1)**
Most favored regions	75%	68.8 %	65.6 %	73.3%	73.3%	94.1 %	76.5 %	70.6 %	58.8 %	73.5 %	72.4%	71.0%
Additional allowed	15.6 %	21.9 %	25.0 %	20%	20%	5.9 %	17.6 %	26.5 %	26.5 %	23.5%	24.1%	29.0%
Generally Allowed	6.2 %	6.2 %	3.1 %	3.3 %	3.3 %	0%	5.9 %	2.9 %	11.8 %	2.9 %	3.4%	0%
Disallowed regions	3.1 %	3.1 %	6.2 %	3.3 %	3.3 %	0%	0%	0%	2.9 %	0%	0%	0%
2nd generation packing quality (correct values >-3)	-4.040 (bad)	-4.153 (bad)	-4.286 (bad)	-4.303 (bad)	-4.165 (bad)	-3.632 (poor)	-3.447 (poor)	-3.229 (poor)	-3.015 (poor)	-3.286 (poor)	-3.720 (poor)	-1.713
Absolute quality of model (correct values: from -1 to 1)	-0.06	-0.306	0.22	-0.797	-0.797	-1.437	-0.855	0.061	-0.846	0.03	N/A	N/A

Table S4. Solutions used in the whole-cell patch/voltage clamp experiments. Concentration values are expressed in mM. Chol-Cl: Choline chloride; TEA-Cl: Tetraethylammonium chloride; 4-AP: 4-Aminopyridine. Intracellular solutions for dorsal root ganglion cells (DRGs) also contained 1 mM Na_2GTP and 2mM MgATP

**Solution**	**KCl**	**NaCl**	**CsCl**	**$CdCl_2$**	**CsF**	**$MgCl_2$**	**$CaCl_2$**	**Chol-Cl**	**KF**	**HEPES**	**EGTA**	**TEA-Cl**	**4-AP**	**pH**
Extracellular I_{Na^+} DRGs		20				1	1.8	70		10		45	10	7.4
Intracellular I_{Na^+} DRGs		10	30		100					5	8	10		7.3
Extracellular I_{K^+} DRGs	10			0.3		1.2	1.8	130		10				7.4
Intracellular I_{K^+} DRGs	50						0.1	60	40	5	10			7.2
Extracellular Snail	4	75				5	7			5				7.4
Intracellular Snail	70	4								20	1			7.3
ND96 solution (oocytes)	2	96				2	1.8			5				7.4
